# Enhanced Histochemical Detection of Iron in Paraffin Sections of Mouse Central Nervous System Tissue: Application in the APP/PS1 Mouse Model of Alzheimer’s Disease

**DOI:** 10.1177/1759091416670978

**Published:** 2016-09-28

**Authors:** Scott A. Sands, Regis Leung-Toung, Yingsheng Wang, John Connelly, Steven M. LeVine

**Affiliations:** 1 Department of Molecular and Integrative Physiology, University of Kansas Medical Center, KS, USA; 2 ApoPharma, Inc., Toronto, Canada

**Keywords:** Alzheimer’s disease, APP/PS1, ferric, histochemistry, iron, plaque

## Abstract

Histochemical methods of detecting iron in the rodent brain result mainly in the labeling of oligodendrocytes, but as all cells utilize iron, this observation suggests that much of the iron in the central nervous system goes undetected. Paraffin embedding of tissue is a standard procedure that is used to prepare sections for microscopic analysis. In the present study, we questioned whether we could modify the iron histochemical procedure to enable a greater detection of iron in paraffin sections. Indeed, various modifications led to the widespread labeling of iron in mouse brain tissue (for instance, labeling of neurons and neuropil). Sites of focal concentrations, such as cytoplasmic punctate or nucleolar staining, were also observed. The modified procedures were applied to paraffin sections of a mouse model (APP/PS1) of Alzheimer’s disease. Iron was revealed in the plaque core and rim. The plaque rim had a fibrillary or granular appearance, and it frequently contained iron-labeled cells. Further analysis indicated that the iron was tightly associated with the core of the plaque, but less so with the rim. In conclusion, modifications to the histochemical staining revealed new insights into the deposition of iron in the central nervous system. In theory, the approach should be transferrable to organs besides the brain and to other species, and the underlying principles should be incorporable into a variety of staining methods.

## Introduction

Iron is an essential element that is utilized by every living cell in the body. In the brain, iron is involved in a multitude of roles that include neurotransmitter synthesis, myelination, respiration, DNA and RNA demethylation, cell proliferation, and so forth (Furukawa et al., 1992; Beard and Connor, 2003; Jia et al., 2008; Westbye et al., 2008; Todorich et al., 2009; Kim and Wessling-Resnick, 2014). Despite this widespread requirement for iron, its distribution in the normal brain has been portrayed by histochemical studies as being highly restricted, with the majority of staining occurring in oligodendrocytes, and their principal product, myelin (Hill and Switzer, 1984; Dwork et al., 1988; Connor and Menzies, 1990; LeVine and Macklin, 1990; LeVine, 1991). During disease, iron has been detected in additional structures; for instance, iron has been shown to be colocalized to plaques (Connor et al., 1992; LeVine, 1997; Smith et al., 1997), which are a hallmark pathological feature of Alzheimer’s disease (AD). Establishing this deposition in plaques is relevant as iron has been implicated in the pathogenesis of AD (Bishop et al., 2002; Castellani et al., 2007; Belaidi and Bush, 2016); however, details regarding the role of iron in disease activity are incomplete.

Performing studies on animal models of neurodegenerative diseases is a widely used approach to advance the understanding of pathogenic mechanisms, but there have been varying results about the association of iron with plaques in mouse models of AD, in particular, the widely used APP/PS1 double transgenic mouse (Falangola et al., 2005; Jack et al., 2005; El Tannir El Tayara et al., 2006; Meadowcroft et al., 2009; Dong et al., 2015; Guo et al., 2015; Meadowcroft et al., 2015). Studies that observed an abundance of iron in plaques often utilized free-floating sections of central nervous system (CNS) tissue for iron histochemical staining (Falangola et al., 2005; Jack et al., 2005; El Tannir El Tayara et al., 2006). Paraffin sections on the other hand appeared to underrepresent the amount of iron deposition in plaques (Dong et al., 2015; Guo et al., 2015). Because paraffin sections are routinely used for pathological analyses, it became relevant to determine whether a protocol for the histochemical detection of iron could be modified to improve the staining of iron in this preparation.

Previous efforts have been made to advance the histochemical detection of iron in paraffin sections (Meguro et al., 2007). Sometimes the staining procedure on paraffin sections utilized tissue processed with an uncommon fixative, that is, methacarn (Smith et al., 1997). One protocol, based on a procedure described by Meguro et al. (2007), was applied to paraffin sections from CNS tissue of AD patients, and it revealed plaque staining (van Duijn et al., 2013). In the present study, we made modifications to this latter procedure and applied it to paraffin sections of APP/PS1 and control mice. Results generated by the modified staining procedure revealed new insights into the association of iron with plaques and provided a new awareness about the distribution of iron in the brain.

## Materials and Methods

### Chemicals

Chemicals used for the staining of tissue sections were as follows: hydrogen peroxide, hydrochloric acid (HCl), methanol, Permount, polyvinylpyrrolidone (PVP) (Cat.#BP431-100), SafeClear Xylene Substitutes, Triton X-100, and potassium ferrocyanide trihydrate (Cat.#S25489) were purchased from Fisher Scientific (Hanover Park, IL). Sodium azide, sodium borohydride, proteinase K, phosphate-buffered saline (PBS), Trizma base, and 3,3′-diaminobenzidine tetrahydrochloride (DAB) (Cat.#D5905) were purchased from Sigma (St. Louis, MO). Chemicals used for test tube studies were as follows: Ferric iron solution (iron Standard for AAS, 1,000 mg/L Fe in nitric acid), sodium azide, and DAB were purchased from Sigma-Aldrich (Oakville, Ontario, Canada), whereas K_4_Fe(CN)_6_ (EM Science), TRIS (EM Science), HCl (BDH), and H_2_O_2_ (BDH) were purchased from VWR (Mississauga, Ontario, Canada). Proper attention to safety is required when handling these chemicals.

### Animals and Tissue Processing

At 12 to 14 months of age, female APP/PS1 (Stock No. 005864, Jackson Lab; *n* = 5) or control (*n* = 9) mice that had been administered vehicle of carboxymethylcellulose (0.5% w/v) via gavage bid for approximately 6 months were anesthetized with isoflurane, and blood was collected via cardiac puncture. This was followed by perfusion with cold saline, removing and dissecting the brain, and immersion fixing it in 4% paraformaldehyde in PBS for 48 hr at 4℃. The tissue was transferred to 70% ethanol for 2 to 3 days at 4℃, and then processed through upgraded alcohols to xylene followed by paraffin. Sagittal sections, 8 µm thick, were prepared, floated on a 38℃ water bath, mounted on a glass slide and air dried (no heating).

### Detection of Ferric Iron Following Deparaffinization, Downgraded Alcohol Steps, and Rehydration

Modifications from the method described in van Duijn et al. (2013), which is based on the method put forth in Meguro et al. (2007), were as follows: (a) including PVP in the potassium ferrocyanide trihydrate and HCl solution, (b) lengthening the incubation time of the potassium ferrocyanide/PVP/HCl solution from 40 to 60 min, (c) dissolving DAB in 0.01 M Tris buffer, pH 7.4, as opposed to 0.1 M phosphate buffer, and (d) increasing the concentration of hydrogen peroxide for the DAB step from 0.005% to 0.12%. The complete procedure is provided below.

Paraffin sections were deparaffinized and processed through downgraded alcohols (SafeClear 2 × 3 min, 100% EtOH 3 min, 100% EtOH 1 min, 95% EtOH 1 min, 90% EtOH 1 min, and 70% EtOH 1 min), rehydrated, and incubated in 1% potassium ferrocyanide trihydrate/5% PVP/0.05 N HCl solution (added as follows: 10 ml of 4% potassium ferrocyanide trihydrate in water, 20 ml of 10% PVP in water, and 10 ml of 0.2 N HCl) for 60 min on a horizontal rotating platform. Sections were rinsed 2 × in water and placed in methanol containing 0.3% hydrogen peroxide and 0.01 M sodium azide for 75 min (van Duijn et al., 2013, which was adapted from Meguro et al., 2007). Sections were then rinsed 2 × in PBS and incubated in a mixture of 10 mg DAB: 40 ml 0.01 M Tris HCl pH 7.4: 160 µl 30% H_2_O_2_ for 40 min. Sections were rinsed 2 × in PBS, dehydrated (reverse of downgraded alcohols and deparaffinization), and coverslipped in Permount. Some sections that were run as controls had potassium ferrocyanide trihydrate eliminated from the incubation mixture.

The effect of acid pretreatment on staining was tested in some sections. Immediately preceding the incubation in 1% potassium ferrocyanide trihydrate/5% PVP/0.05 N HCl, sections were incubated in 0.05 N HCl for 30 min × 2 and then stained as described earlier. Additionally, a subset of sections was processed to test the effect of protease digestion on iron staining using a method modified from LeVine (1991). Immediately after deparaffinization, downgraded ethanols, and rehydration, sections were washed in PBS and incubated in 10 mg sodium borohydride (Sigma)/ml PBS for 30 min. Sections were washed in PBS, incubated in 30 µg proteinase K (Sigma)/ml PBS with 0.1% Triton X-100 (Fisher Scientific) for 20 min, washed in PBS, and then processed as described earlier starting with the incubation in the staining solution. To compare the effects of this treatment, matched sections were run in parallel but in place of the sodium borohydride and proteinase K steps, sections remained in PBS, and were then processed equivalently. In a blinded manner, cortical plaques were counted from four pictures per mouse (taken with a 20 × objective on a Nikon Eclipse 80i microscope with an Olympus DP72 digital camera and Olympus cellSens standard imaging software) of matched pairs of slides (with or without proteinase K) from three APP/PS1 mice.

### Detection of Ferric Iron Without Deparaffinization, Downgraded Alcohol Steps, and Rehydration

In place of deparaffinization, downgraded ethanols, and rehydration, paraffin sections were directly placed in the potassium ferrocyanide/PVP/HCl staining solution and processed as described earlier for ferric iron staining. To examine the effect of brief deparaffinization (without subsequent treatment with downgraded alcohols and rehydration) on staining characteristics, some sections were incubated in SafeClear 2 × 3 min, air dried, then processed as described earlier starting with the potassium ferrocyanide/PVP/HCl solution.

### Effect of Sodium Azide on Staining

A feature in the procedure put forward by Meguro et al. (2007) is an incubation step in a solution of sodium azide and hydrogen peroxide in methanol following the potassium ferrocyanide/HCl incubation but before the DAB step (see earlier). The rationale provided for this step was to block endogenous catalase, peroxidase, and pseudoperoxidase activities (Meguro et al., 2003). However, we questioned whether in addition to these functions, the sodium azide acted on the Prussian blue precipitant (product from ferric iron reacting with potassium ferrocyanide/HCl) to enable it to amplify the DAB staining. To test for these effects, a solution of 1.30 e^−4 ^M Fe^3+^/9.80 e^−5 ^M K_4_Fe(CN)_6_/0.05 N HCl was sonicated for 20 min, and 5 g of solution was added to either 1000 μl of 30% H_2_O_2_/100 ml of MeOH (no azide; with hydrogen peroxide) or 65 mg of NaN_3_/1000 μl 30% H_2_O_2_/100 ml of MeOH (with azide; with hydrogen peroxide) to a total volume of 25 ml and the resulting solutions were sonicated for 75 min. In separate tubes, either of these latter solutions was added to 22 g of DAB solution to bring the volume to 25 ml. The DAB solution was prepared as follows: 12.5 mg DAB/48 ml of 0.01 M Tris, pH 7.4, was sonicated for 40 min and then 200 μl of 30% H_2_O_2_ and 0.01 M Tris pH 7.4 were added to bring the volume to 50 ml and filtered through a 45 µm high-performance liquid chromatography filter.

An additional experiment examined the effect of sodium azide or hydrogen peroxide on staining features. Some paraffin sections processed with or without deparaffinization, downgraded ethanols, and rehydration were processed as described earlier for ferric iron staining, except sodium azide or hydrogen peroxide (matched pairs) was eliminated from the methanol solution and the incubation in the DAB solution was shortened to 20 min for the sections processed without deparaffinization, downgraded ethanols, and rehydration but kept at 40 min for sections with these steps.

### Effect of PVP on Staining

Some paraffin sections processed with or without deparaffinization, downgraded ethanols, and rehydration were further processed as described earlier for ferric iron staining, except in matched pairs of sections in which PVP was replaced with water or retained in the solution of 1% potassium ferrocyanide trihydrate/5% PVP/0.05 N HCl.

### Effect of Direct Staining on Free-Floating Sections

Some mice, approximately 2.5 months of age, were deeply anesthetized with isoflurane and perfused cardially with ice cold PBS followed by cold 4% paraformaldehyde. Brains were immersion fixed in 4% paraformaldehyde at 4℃, 24 to 48 hr, and then transferred to a cryoprotectant solution of 25% glycerol/2.5% DMSO in PBS at 4℃ for ≥24 hr. Frozen microtome sections, 50 µm, were prepared and placed directly in water or PBS on a rotating platform, 2 × 15 min, and then processed for staining of ferric iron as described earlier, except the DAB step was 20 min. Additional frozen microtome sections were placed directly in the potassium ferrocyanide trihydrate/PVP/HCl staining solution in place of water or PBS and then processed for staining as described earlier except the DAB step was 20 min.

## Results

**Figure 1.**
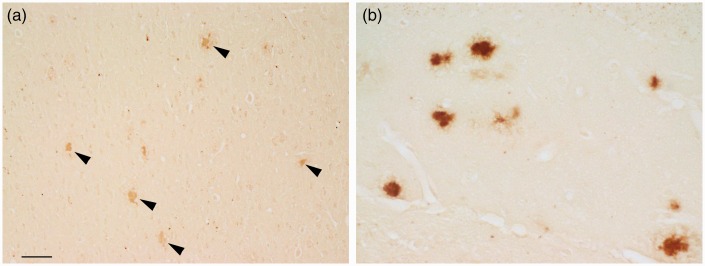
Following deparaffinization, downgraded ethanols, and rehydration, the modified iron histochemical stain revealed plaque staining in the (a) cortex, (b) hippocampus, and in other CNS structures in APP/PS1 mice. The dense core was the site of most concentrated stain within the plaque. Bar in a = 60 µ (a) and 20 µ (b). *Note*. CNS = central nervous system.

Traditional processing of paraffin-embedded sections utilizes deparaffinization, graded alcohols, and rehydration steps prior to staining. The ferric iron that remains after these steps, and positioned to react with the staining reagents, is discretely localized within the sagittal sections. In APP/PS1 mice, plaques were most often observed to have dense core staining, but sometimes diffuse labeling was also present. Plaques were present in the cortex (Figure 1(a)), hippocampus (Figure 1(b)), cerebellum, and less often in the caudate putamen and thalamic nuclei.

**Figure 2.**
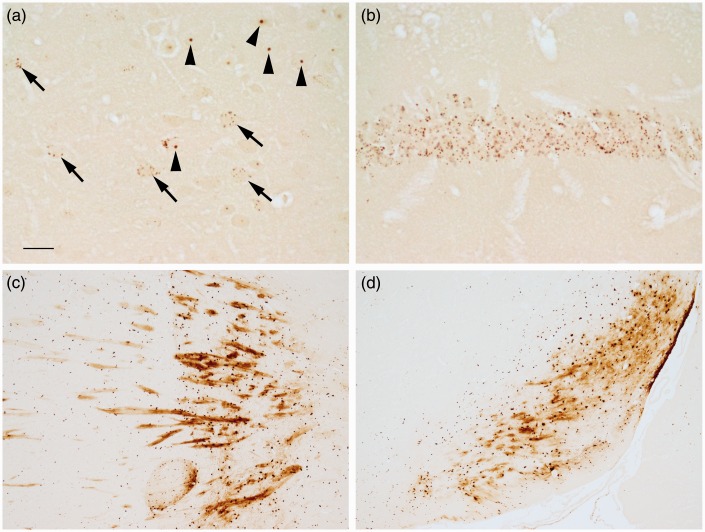
The modified iron histochemical staining following deparaffinization, downgraded ethanols, and hydration revealed staining in discrete structures. (a) Punctate (arrows) and nucleolus (arrowheads) staining was observed in some cells, presumably neurons, in the cortex. (b) Punctate staining was observed in the hippocampal CA neuronal layer, particularly the CA1 region. (c) Myelinated fibers and oligodendrocytes were labeled in the globus pallidus (right/center) and the caudate or putamen (left). (d) Myelin and oligodendrocytes were labeled in the substantia nigra. Control mice shown (a–d). Bar in a = 20 µ (a and b) and 120 µ (c and d). *Note*. CA = Cornu Ammonis.

In addition to plaques, other structures were stained, which were usually present in both APP/PS1 and control mice. Punctate staining was present in neurons in the CA hippocampal layer and the dentate gyrus, with labeling being the most pronounced in CA1 neurons (Figure 2(b)), and sometimes these cells had staining in the nucleus, nucleolus, and/or cytoplasm. Punctate labeling was also present within some cells, which appeared to be neurons, in the cortex (Figure 2(a)). Besides punctate labeling, occasionally cells had cytoplasmic, nuclear, and/or nucleolar (Figure 2(a)) staining. Myelin and oligodendrocyte staining was present predominantly in the globus pallidus, ventral pallidum, caudate putamen, and substantia nigra (Figure 2(c) and (d)). Additionally, some ependymal cells and aggregates of staining around the ventricle were observed, and some labeled cells were present in the thalamus, cerebellar nuclei, and cerebellum (not shown). Of note, these staining features and plaque staining were amplified in one section that was inadvertently made thicker, that is, 10 µm instead of 8 µm, suggesting that section thickness can influence staining density.

**Figure 3.**
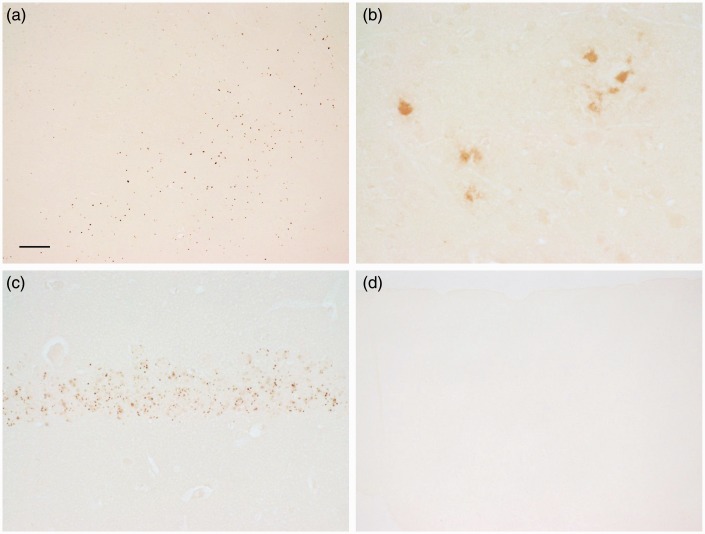
Sections that underwent deparaffinization, downgraded ethanols, and rehydration were treated with (a–c) HCl prior to staining or (d) had potassium ferrocyanide not included in the staining solution. (a) HCl removed labeling of myelin (control mouse), but some staining of cells remained in the globus pallidus (right/center). Staining of (b) the dense core of plaques (APP/PS1 mouse) and (c) puncta in hippocampal CA1 neurons (control mouse) remained following pretreatment with HCl. (d) Removal of potassium ferrocyanide from the staining solution eliminated staining (cortex is shown with the pial surface at the top; APP/PS1 mouse). Bar in a = 120 µ (a and d) and 20 µ (b and c). *Note*. HCl = hydrochloride.

**Figure 4.**
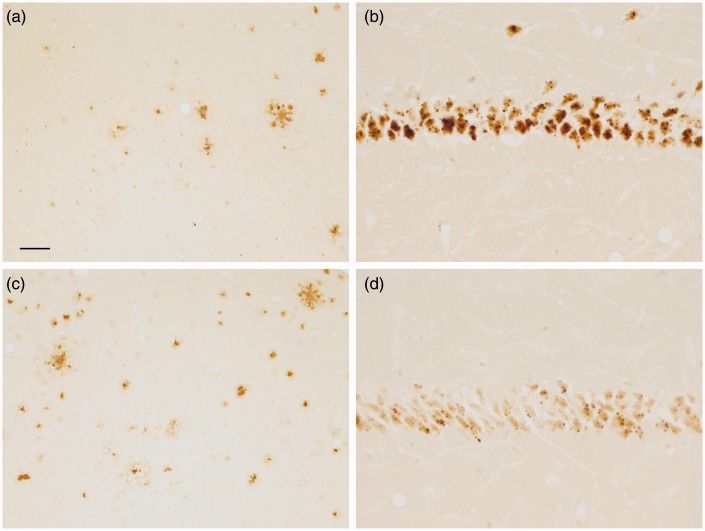
Sections that underwent deparaffinization, downgraded ethanols, and rehydration were treated with (a and b) PBS or (C and D) sodium borohydride and proteinase K with Triton X-100 prior to staining. In comparison to (a) PBS, (c) proteinase K appeared to uncover plaque staining in the cortex (matched pair of sections from the same APP/PS1 animal). In comparison to (b) PBS, (d) proteinase K removed or lessened some of the nuclear and punctate staining that was present in hippocampal CA1 neurons (matched pair of sections from the same control animal). Bar in a = 60 µ (a and c) and 20 µ (b and d). *Note*. PBS = phosphate-buffered saline.

Leaving out potassium ferrocyanide trihydrate from the staining procedure eliminated the staining of the structures described earlier (Figure 3(d)). Additional sections were pretreated with HCl prior to incubation in potassium ferrocyanide/PVP/HCl. This pretreatment eliminated or reduced much of the staining across the section (Figure 3(a)). The primary exceptions were the dense cores of plaques (Figure 3(b)) and some punctate structures, for example, present within CA1 hippocampal neurons (Figure 3(c)). The retention of labeling within the plaque core following HCl treatment suggested that some iron is tightly associated or deeply embedded within the core. To evaluate whether proteinaceous material was masking some of the iron in plaques, additional sections were pretreated with sodium borohydride followed by proteinase K in a Triton X-100 solution before being processed for the rest of the staining protocol. Digested sections revealed a greater labeling (increased plaque numbers for each of the three matched slide pairs) of plaques in APP/PS1 mice compared with no proteinase K treatment (Figure 4(a) and (c)), while staining of other structures was eliminated or reduced (Figure 4(b) and (d)).

**Figure 5.**
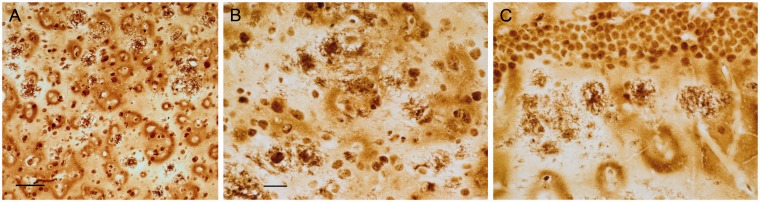
Sections from APP/PS1 mice that were stained directly (i.e., they did not undergo deparaffinization, downgraded ethanols, and rehydration) revealed labeling of plaques among the splotches of staining in the (a and b) cortex and (c) hippocampus. Besides staining of the core, fibrillary and/or amorphous staining was associated with the plaque. Staining of nuclei was also abundant. Bar in a = 60 µ (a). Bar in b = 20 µ (b and c).

As deparaffinization followed by downgraded ethanol steps to hydration is thought to extract loosely associated iron, sections were processed directly for staining without any steps to first remove paraffin. Extensive labeling of plaques (in number, staining intensity, and varying sizes) was revealed. Often the plaque staining appeared as a fibrillary network with or without dense core staining (Figure 5), with the thin fibrillary structures often extending into the neuropil. Labeled cells were often observed within the rim of plaques. Some plaques also had diffuse staining. This high level and detail of staining within plaques was greater than that revealed from staining following deparaffinization, downgraded ethanols, and rehydration steps (Figure 1). Areas of uneven staining (e.g., splotches) were also present throughout much of the sections, but the staining of the plaques was distinct, especially at higher magnification (Figure 5).

**Figure 6.**
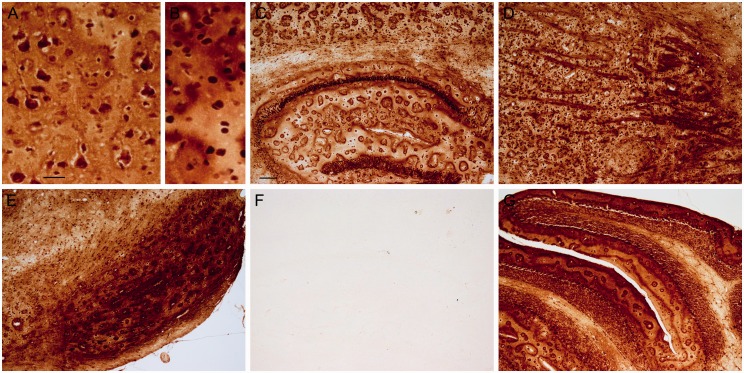
Sections from control mice that underwent direct staining (i.e., no deparaffinization) revealed staining of nuclei (and sometimes distinct cytoplasmic staining) of neurons, myelin, and neuropil staining. (a and b) Staining in the cortex is revealed in nuclei, cytoplasm, and neuropil. (c) The hippocampal CA1 neurons are densely stained. Splotches of staining are apparent in the cortex and hippocampus. (d) Myelin staining in the globus pallidus (right) and caudate or putamen (left) is apparent among staining of cells (dark dots) and neuropil across the field. Some splotches of staining are also apparent (lower left). (e) Dark staining of myelin and neuropil is present in the substantia nigra. Staining of cells (dark dots) across the section is also apparent. (f) Eliminating the potassium ferrocyanide from the staining solution reveals an absence of labeling. Hippocampus is shown (compare to c). Note, some tissue artifact (upper right) is lightly labeled showing that the section was exposed to the DAB solution, and an extremely light depiction of the CA1 neurons can be made out. (g) Staining of the cerebellum reveals staining of granule cells, Purkinje neurons, and staining of neuropil and neurons in the molecular layer, which also has a heterogeneous pattern of staining. Bar in a = 20 µ (a,b). Bar in c = 100 µ (c–g). *Note*. DAB = 3,3′-diaminobenzidine tetrahydrochloride.

Besides plaques, distinct staining of nuclei was present throughout the section and often details within the nuclei, for example, nucleoli, were apparent (Figure 6(a) and (b)). The nuclei of CA neurons in the hippocampus and neuropil in the stratum oriens and radiatum were labeled (Figure 6(c)). In addition, staining of myelin was apparent in the caudate or putamen, globus pallidus, and substantia nigra (Figure 6(d) and (e)). The staining of the neuropil in the molecular layer of the cerebellum stood out compared with background staining in the other layers, although granule cells, Purkinje neurons, and cells within the white matter were also stained (Figure 6(g)). Removing the potassium ferrocyanide trihydrate from the staining mixture prevented the staining of the above structures (Figure 6(f)).

**Figure 7.**
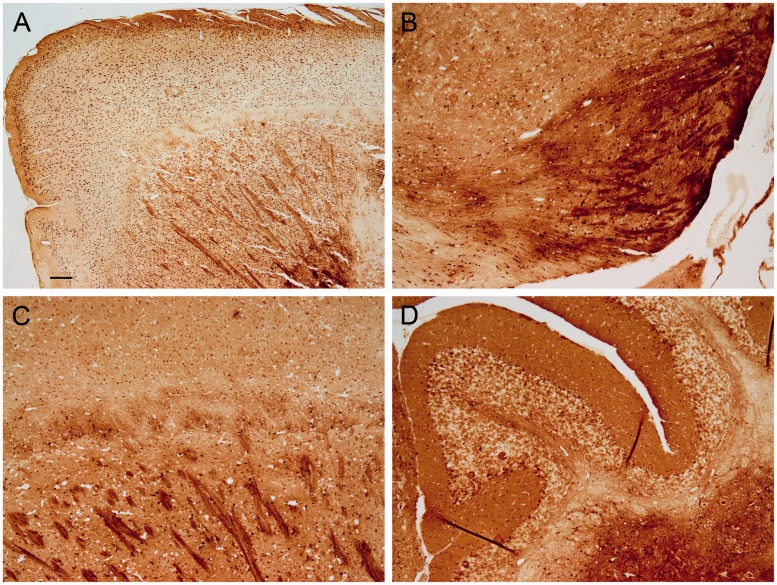
Sections from control mice were treated with SafeClear followed by air drying prior to staining. Much of the splotchy or uneven staining observed in Figures 5 and 6 (no SafeClear) is removed, but staining is still much darker than that observed in Figure 2 (deparaffinization, downgraded ethanols, and rehydration prior to staining). (a) Staining of the frontal cerebrum reveals labeling of cells throughout the cortex and caudate or putamen, which also had myelin staining. (b) Labeling of cells is apparent in the substantia nigra and adjacent structures. Myelin and dark neuropil staining is apparent in the substantia nigra. (c) Some staining of the corpus callosum (horizontal band of uneven staining) is apparent above the myelin staining in the caudate or putamen. Labeled cells are apparent across the field. (d) Labeling of the neuropil in the molecular layer of the cerebellum is relatively uniform and distinct. Labeling of some myelin fibers (e.g., in the tips of the folia), granule cells, and Purkinje cells is also apparent. Bar in a = 200 µ (a) and 80 µ (b–d).

**Figure 8.**
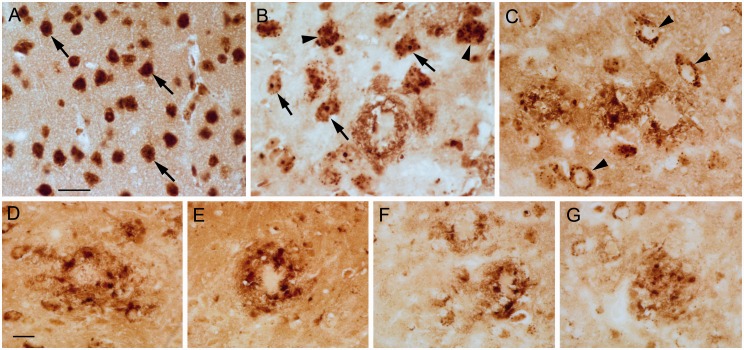
Treatment of sections with SafeClear followed by air drying prior to staining generated greater details of staining compared to sections processed without SafeClear or sections that underwent deparaffinization, downgraded ethanols, and hydration. (a) The soma, nucleus, and nucleolus (see cells at arrows) are apparent in neurons in the cortex of a control mouse. Note the heterogeneous staining within the nucleus. Smaller, lighter stained cells are also labeled. (b) Punctate staining within the soma (arrowheads) was apparent, which was sometimes also present with nucleolus labeling (arrows). A plaque with rim staining is present. (c) Sometimes cells had light labeling of the nucleolus/nucleus, but prominent labeling of puncta in the cytoplasm (see cells at arrowheads). Labeling of plaques is also present. (d–g) Examples of plaques associated with intensely labeled cells that were usually present within the plaque rim (d–f). Bar in a = 20 µ (a–c). Bar in d = 20 µ (d–g). Control (a) and APP/PS1 (b–g) mice.

**Figure 9.**
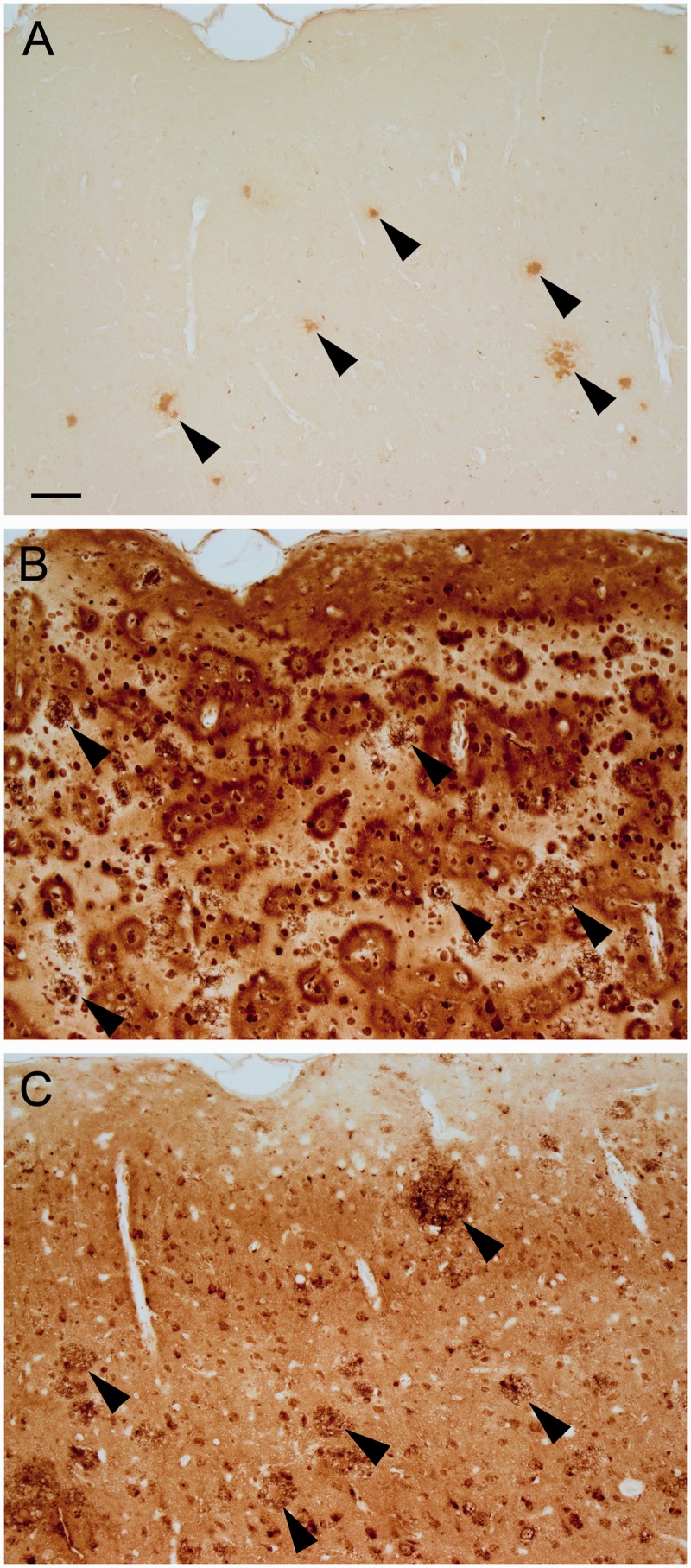
Matched sections from a APP/PS1 mouse underwent (a) deparaffinization, downgraded ethanols, and hydration, (b) direct staining, or (c) SafeClear followed by air drying. Plaques in the cortex (arrowheads) were revealed with all three conditions, but (a) revealed staining that was predominantly limited to the core. More detailed features of plaque were labeled in (b) and (c). Cell labeling was also present throughout the cortex in (b) and (c), but (c) did not have the splotches of staining that were observed in (b) although staining was lighter in (c) than for (b). Bar in a = 50 µ (a–c).

Deparaffinization of the tissue with xylenes for 10 min directly followed by drying does not alter the iron concentration, measured analytically, compared with fresh samples (Olynyk et al., 1994). Thus, we treated some sections with SafeClear (2 × 3 min), air dried, and then processed the sections for iron staining. The generalized neuropil (or background) staining was more uniformly distributed across the section than without any deparaffinization, and the neuropil in the globus pallidus and ventral pallidum had more definition (Figure 7). Also, neuropil staining in the molecular layer became more distinct, but the staining of cerebellar granule cells lightened (Figure 7(d)). The staining of iron in plaques persisted with staining present in the cores, fibrillary network, and dysmorphic cells within the plaque rim (Figures 8 and 9(c)). However, the staining was lighter than without SafeClear but still considerably more intense than that found in sections that underwent deparaffinization followed by downgraded alcohol steps to hydration (Figure 9) suggesting substantial extraction of stainable iron by these latter processing steps. In general, the nuclear staining lightened, but the staining of the soma, which was often granular or punctate, was revealed (Figure 8(a)–(c)). In some instances, there was a dense accumulation of staining present in the nucleolus (Figure 8(a) and (b)). Interestingly, the staining of myelin became more distinct by the inclusion of SafeClear with air drying. Not only was the staining of myelin more distinct in the caudate or putamen, globus pallidus, and substantia nigra, but staining in the subcortical white matter (e.g., posterior corpus callosum) and in the tips of the white matter in the cerebellar folia became more apparent (Figure 7), although to a lesser, more variable, and lighter degree in the latter.

**Figure 10.**
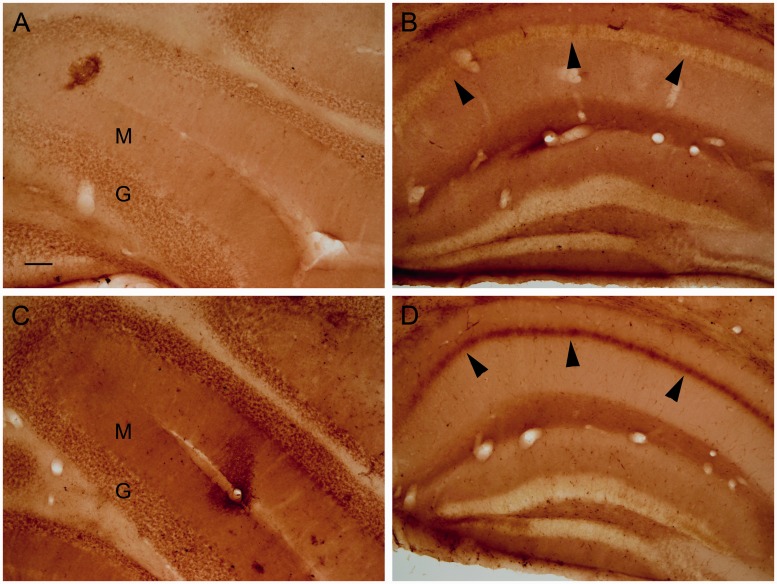
Frozen sections from approximately 2.5-month-old control animals were (a and b) placed in water and then stained, or (c and d) directly stained upon sectioning. The molecular (M) and granule cell (G) layers of the cerebellum were darker in the directly stained section (c) compared with the section placed in water prior to staining (a). The hippocampal CA1 neuron layer (arrowheads) had greater labeling in the section that was directly stained (d) versus incubating the section in water prior to staining (b). Bar in a = 100 µ (a–d).

To have a second test to determine whether some iron can be extracted during processing, upon sectioning, free-floating frozen sections were placed directly in the potassium ferrocyanide/PVP/HCl staining solution, while other sections were placed first in water or PBS, washed, and then stained. Placing the section directly into the staining solution typically gave more pronounced labeling, for example, within the CA1 neurons in the hippocampus and within the molecular and granule cell layers in the cerebellum, in comparison to exposing the sections to water, and to a lesser degree PBS, prior to staining (Figure 10).

**Figure 11.**
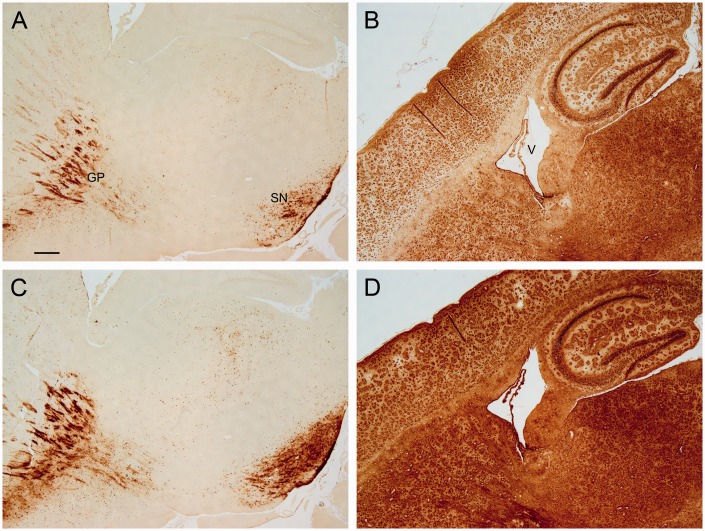
Matched sections from control animals were processed with the (a and b) absence or (c and d) presence of PVP in the staining solution. For sections processed with deparaffinization, downgraded ethanols, and hydration, the staining was greater in the presence of PVP (c) compared with the absence of PVP (a). Note the greater labeling at the GP and the SN for sections stained with PVP. For sections that were directly stained, PVP gave greater labeling across the section (d) compared with no PVP (b) (V, ventricle). Bar in a = 250 µ (a–d). *Note*. GP = globus pallidus; SN = substantia nigra; PVP = polyvinylpyrrolidone.

To test the effects of PVP on staining, it was removed from the staining solution leaving just potassium ferrocyanide/HCl. Staining was greater in the presence of PVP than in its absence, with or without deparaffinization, downgraded alcohols, and hydration (Figure 11).

**Figure 12.**
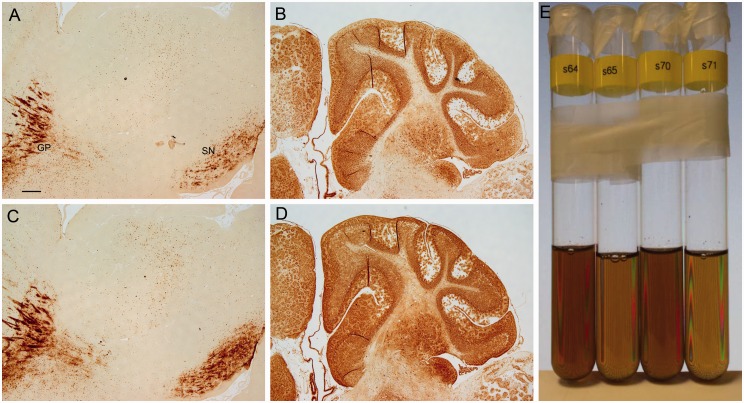
Matched sections from control animals were processed with the (a and b) absence of azide (with hydrogen peroxide) or (c and d) in the presence of azide (no hydrogen peroxide) in methanol. Sections that were processed with deparaffinization, downgraded ethanols, and hydration gave greater staining with azide (c) compared with the absence of azide (a). The labeling at the GP and the SN was greater in sections processed with azide. In sections that were directly stained, azide resulted in greater labeling of cerebellar structures (d) compared with no azide (b). Bar in a = 250 µ (a–d). (e) Solutions containing ferric iron, potassium ferrocyanide, HCl, hydrogen peroxide with (Tubes 64 and 70) and without (Tubes 65 and 71) azide were incubated with DAB for 1 hr (see Methods section). Solutions with azide gave a darker brown solution than solutions without azide. *Note*. GP = globus pallidus; SN = substantia nigra; DAB = 3,3′-diaminobenzidine tetrahydrochloride.

To test the effects of sodium azide on staining, sodium azide or hydrogen peroxide was removed from the methanol solution. Overall, staining intensity was greater in the presence of sodium azide (without hydrogen peroxide), than in the absence of sodium azide (with hydrogen peroxide; Figure 12). This was observed for paraffin sections processed with or without deparaffinization, downgraded alcohols, and hydration (Figure 12), although this difference in staining was more pronounced for the latter condition, that is, without these steps. To validate observations from stained sections, additional studies tested whether sodium azide increased the reactivity of Prussian blue to catalyze the oxidation of DAB (i.e., formation of brown color) in test tubes. Indeed, the presence of sodium azide resulted in a darker brown solution than in the absence of sodium azide (Figure 12(e)).

## Discussion

Iron is an essential element that is used for biochemical reactions by all cells in the brain. However, standard histochemical methods for detection of iron typically reveal its presence in a limited set of structures, with the oligodendrocyte and myelin being the most commonly labeled entities (Hill and Switzer, 1984; Dwork et al., 1988; Connor and Menzies, 1990; LeVine and Macklin, 1990; LeVine, 1991). Thus, traditional histochemical procedures fail to reveal a comprehensive portrayal of the distribution of iron in the CNS. To increase the sensitivity for the detection of iron, histochemical procedures have undergone multiple modifications. The Perls histochemical stain dates back to 1867 (Perls, 1867), and it is still used today. This stain incubates the tissue in a solution of HCl and potassium ferrocyanide, which interacts with ferric iron in the tissue to form a Prussian blue precipitant (Meguro et al., 2007). The ability of the Perls stain to detect low amounts of iron is limited compared with analytical methods (Barry, 1974; van Deursen et al., 1988a). In 1980, Nguyen-Legros et al. reported that DAB and hydrogen peroxide can increase staining. In particular, the Prussian blue precipitant, generated by the Perls stain, catalyzes the oxidation of DAB by hydrogen peroxide, forming an insoluble brown precipitant (Nguyen-Legros et al., 1980). Further intensification could be achieved by lengthening incubation steps and/or increasing the concentration of reagents (Smith et al., 1997; Fukunaga et al., 2010), increasing the permeability of the sections to the staining reagents (LeVine, 1991; 1997), or by using a DAB-enhanced Turnbull stain of brain sections exposed to ammonium sulfide to enable the labeling of both ferrous and ferric nonheme iron (Bagnato et al., 2011; Hametner et al., 2013; Haider et al., 2014). These modifications can lead to the labeling of cells and structures other than oligodendrocytes and myelin, particularly in pathological states. Meguro et al. (2007) described a protocol that incubated the tissue sections in a methanol solution containing sodium azide and hydrogen peroxide. The incubation occurred between the Perls’ stain and DAB intensification step, and it was implemented to inactivate endogenous peroxidases and catalase (Meguro et al., 2003; 2007). In the present study, we show that sodium azide interacts with the Prussian blue precipitant making it more active in catalyzing the oxidation of DAB in the presence of hydrogen peroxide.

Unlike staining obtained with more standard iron histochemical protocols, modifications utilized in the current protocols revealed that iron was extensively distributed in cells and various structures (e.g., neuropil and/or myelin) throughout paraffin brain sections. A broad distribution of iron in the brain is supported by findings using the particle-induced X-ray emission (PIXE) technique, which detected iron in an extracellular location and within the cytoplasm (e.g., punctate staining), nucleus, and nucleolus of neurons in paraffin sections of the rat CNS (Fiedler et al., 2007). A key modification to the protocol that led to the present histochemical results was the elimination of deparaffinization, downgraded alcohols, and hydration steps.

Multiple studies have examined whether formalin fixation or embedding tissue in paraffin alters the concentration of iron compared with that observed in fresh tissue. Although long-term fixation of whole brain in formalin resulted in an increase of iron concentration compared with fresh material, fixation for 18 months or less resulted in comparable levels between fixed and fresh tissue (Gellein et al., 2008). Additionally, one study did observe an increase of iron in formalin-fixed, paraffin-embedded tissue compared with frozen or fresh tissue, but the increase could not be attributed to the formalin or paraffin processing and was left unexplained (Bischoff et al., 2008). In contrast, numerous analytical studies found that fixation or paraffin processing, without downgraded ethanols to hydration, does not appreciably affect the concentration of iron in tissue or it results in a lessening of its concentration (Barry, 1974; Chua-anusorn et al., 1997; van Deursen et al., 1988b; Olynyk et al., 1994; Beilby et al., 1999; Fiedler et al., 2007; Sarafanov et al., 2008; Schrag et al., 2010). Thus, in the present study, the widespread distribution of iron revealed by eliminating deparaffinization, downgraded alcohols, and hydration steps likely depicts iron that originated within the tissue. If sections were deparaffinized with SafeClear and then air dried, staining across the section became more evenly distributed than without these steps, and some structures became more defined while others were lighter. Therefore, a case could be made to include a deparaffinization step followed by air drying prior to staining.

The loss of staining in paraffin sections that underwent deparaffinization, downgraded alcohols, and hydration indicates that much of the iron is readily extractable or becomes undetectable. The possibility that some iron is extractable was confirmed by different treatments of free-floating sections of frozen fixed tissue. For instance, sections of fixed brain tissue that were placed directly in the staining solution typically gave greater labeling compared with sections that were exposed to an aqueous solution prior to staining. Therefore, a portion of the iron within the sections (either free floating or paraffin) is not tightly bound within the fixed tissue. In theory, this iron could be part of the labile iron pool, which has the capacity to catalyze the formation of reactive oxygen species (Kakhlon and Cabantchik, 2002). However, some iron does remain following deparaffinization, downgraded ethanols, and hydration, for example, in plaques and in oligodendrocytes and myelin in the substantia nigra and globus pallidus in paraffin sections. Furthermore, a percentage of this iron appears to be tightly embedded within the tissue as it was stained following exposure to HCl or proteinase K. This was particularly true for iron within plaque cores in paraffin sections where proteinase K treatment suggested an increased number of labeled plaques, while other structures, such as myelin, lost staining.

Amyloid β has multiple residues that bind iron, and this process may promote the aggregation of amyloid β, especially at a pH of 6.6 or 6.8 (Atwood et al., 1998; Castellani et al., 2007). However, it is unclear whether the association of iron with amyloid β is deleterious or protective relative to disease pathogenesis (Rottkamp et al., 2002; Castellani et al., 2007). Besides being enriched within the core, iron deposition was observed in the rim of plaques, particularly, when deparaffinization, downgraded ethanols, and hydration were eliminated from the procedure or when SafeClear was followed by air drying. In comparison to the iron concentration in the neuropil, the concentration of iron was increased in both the rim and core of plaques when detection was done using PIXE (Lovell et al., 1998). Our results suggest that in contrast to the core, the iron in the rim is less tightly associated with plaque constituents as it was not consistently apparent in sections that underwent deparaffinization, downgraded ethanols, and hydration. Interestingly, patients with AD have been found to have higher levels of loosely associated iron than control subjects, and therefore, it was suggested that the level of redox active iron was elevated in AD (Kala et al., 1996). The present results raise the possibility that some of the higher levels of loosely associated iron are from plaque rims where it is potentially more redox active. Thus, it is conceivable that the association of iron with amyloid β may be positioned to be either protective (e.g., within the plaque core) or deleterious (e.g., associated with the plaque rim). Interestingly, stained cells were often observed within the rim of plaques, but the identity and status (e.g., degenerating) of these cells are unknown.

Besides plaques, staining was often observed as punctate staining within neurons, and staining was observed within the nucleus and sometimes within the nucleolus. A PIXE study observed that iron within the cytoplasm and nucleus can be punctiform and that the nucleolus is a site of concentrated iron (Fiedler et al., 2007). Previous histochemical studies have described nucleolar (Sukhorukova et al., 2013) or cytoplasmic punctate staining, which was attributed to iron within lysosomes and/or mitochondria (Meguro et al., 2008). Additionally, in free-floating sections, some cells had unusual accumulations of punctate staining in CNS tissue from patients with AD and to a lesser degree in CNS tissue from patients with multiple sclerosis, and it was suggested that this abnormal punctate staining represented cells undergoing stress (LeVine, 1997). In the present study, extensive punctate staining could be observed in both normal and APP/PS1 neurons, particularly in sections that underwent SafeClear followed by air drying prior to staining. However, a more comprehensive analysis than that performed in the present study is needed to establish whether changes in cytoplasmic iron deposition occur in APP/PS1 neurons, and if so, if it is an indicator of stressed cells that are headed toward neurodegeneration.

In summary, much of the iron present in brain sections goes undetectable by standard histochemical staining methods. We show that iron can diffuse out of the tissue prior to staining giving rise to an underrepresentation of iron deposition. Eliminating deparaffinization, downgraded ethanols, and hydration steps, or processing the sections only through SafeClear followed by air drying prior to staining, can lead to a substantial increase in the detection of stainable iron. As paraffin embedding is a routine method of preparing tissue for sectioning, the modified method presented here could be applicable to specimens from various organs and various species; however, optimization and/or further modifications might be necessary. Furthermore, it is anticipated that in addition to iron, a similar modified approach can be applied to the detection of other compounds.
